# Diagnosis of reactive lymphoid hyperplasia of the bile duct observed on peroral video cholangioscopy

**DOI:** 10.1055/a-2638-5631

**Published:** 2025-07-15

**Authors:** Nami Miyamoto, Masaya Iwamuro, Kiyoaki Ochi, Yosuke Saragai, Tsuneyoshi Ogawa, Toru Ueki

**Affiliations:** 1 13757 Department of Internal Medicine, Fukuyama City Hospital, Fukuyama, Japan; 2 Department of Gastroenterology and Hepatology, Okayama University Graduate School of Medicine, Dentistry, and Pharmaceutical Sciences, Okayama, Japan

A 74-year-old Japanese woman presented with epigastric discomfort and elevated gamma-glutamyl transpeptidase levels (76 U/L). Abdominal ultrasonography revealed gallstones and bile duct wall thickening, prompting referral to our hospital. Contrast-enhanced computed tomography confirmed common bile duct dilation and wall thickening ( Fig. 1 ), while magnetic resonance imaging revealed right hepatic duct dilation. Endoscopic ultrasonography detected hyperechoic areas in the common bile duct and gallbladder, suggesting stones or debris. Endoscopic retrograde cholangiopancreatography confirmed choledocholithiasis, and stones were removed. Intraductal ultrasound identified multiple hypoechoic, subepithelial lesion-like protrusions with hyperechoic margins in the right hepatic duct ( Fig. 2 ). Peroral cholangioscopy under carbon dioxide (CO _2_ ) insufflation revealed multiple subepithelial lesions with dilated surface vasculature ( Fig. 3 , Video 1 ). Furthermore, forceps biopsy demonstrated intact mucosa with lymphoid hyperplasia, without neoplastic changes. Subsequent immunohistochemical staining detected mixed CD20 and CD3 expression, prompting a diagnosis of reactive lymphoid hyperplasia (RLH) ( Fig. 4 ). No specific treatment was pursued for the biliary RLH. Laparoscopic cholecystectomy was performed to address symptomatic gallstones; however, RLH was absent in the resected gallbladder.

**Fig. 1:**
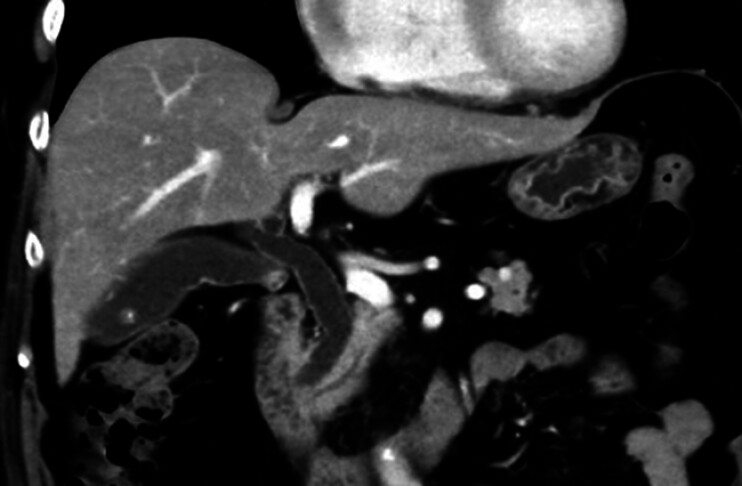
Contrast-enhanced computed tomography showed common bile duct dilation and mild wall thickening.

**Fig. 2:**
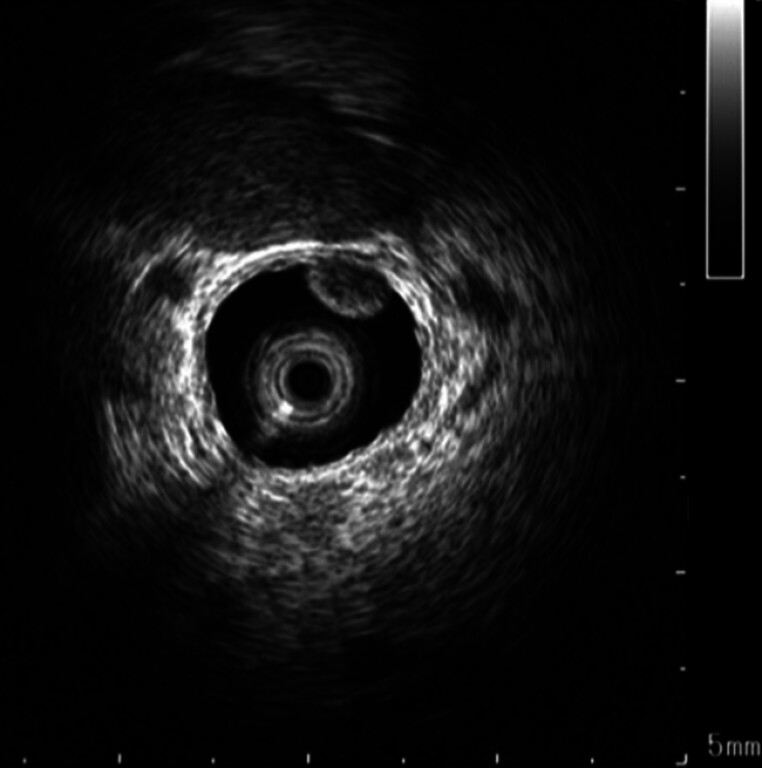
Intraductal ultrasound showed multiple hypoechoic, subepithelial lesion-like protrusions with hyperechoic margins in the right hepatic duct.

**Fig. 3:**
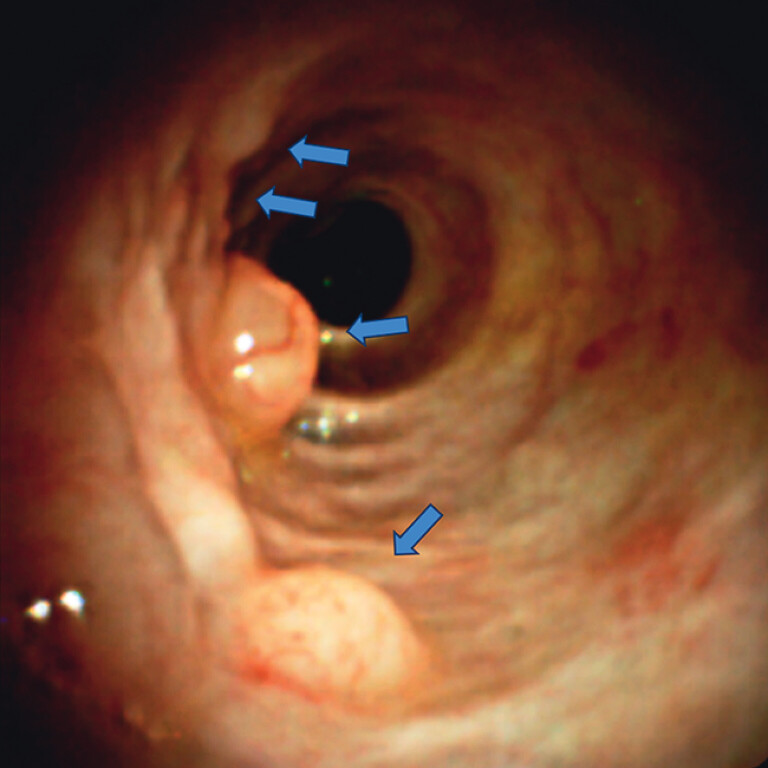
Peroral cholangioscopy revealed multiple subepithelial lesions (arrows) with dilated vasculature on the surface.

**Fig. 4:**
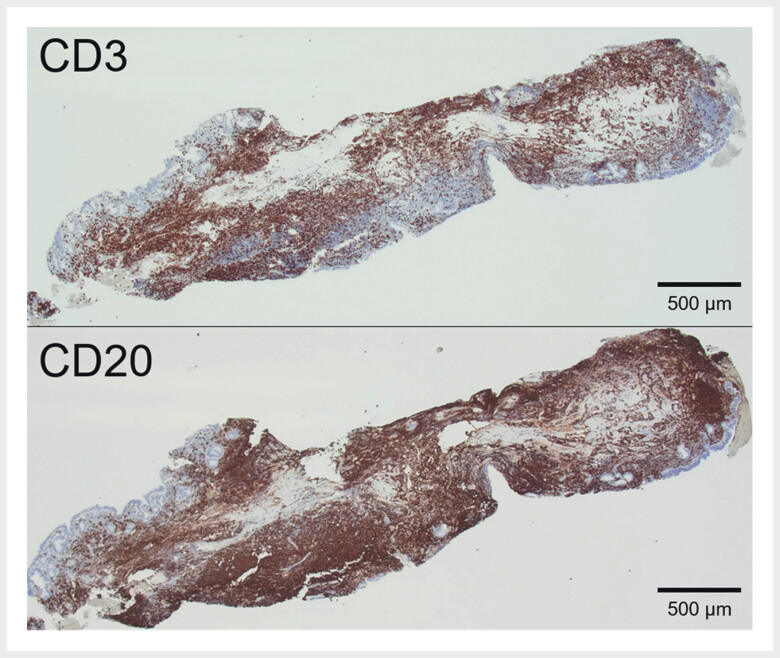
Immunohistochemistry staining showed a clear distinction between CD3, which is positive in T-cell lymphoma, whereas CD20 is positive in B-cell lymphoma, leading to the exclusion of malignant lymphomas other than follicular lymphoma.

Peroral cholangioscopy under carbon dioxide insufflation. Reactive lymphoid hyperplasia appeared as multiple subepithelial lesions with dilated surface vasculature on cholangioscopy.Video 1

Biliary RLH is rare and is believed to be associated with inflammatory conditions such as cholelithiasis and cholangitis, as well as malignancies. To our knowledge, only three cases of biliary RLH have been reported 1 2 3 . Among them, peroral cholangioscopy was performed in two cases using saline irrigation, revealing villous and granular lesions in one case 1 and a polypoid lesion in the other 3 . In contrast, our case exhibited submucosal tumor-like protrusions with dilated vasculature, a finding that is distinct from those previously reported. CO _2_ replacement may have contributed to clearer visualization of the polypoid lesion, consistent with previous studies that indicated superior imaging performance with CO _2_ compared with saline irrigation 4 . This case highlights the utility of peroral cholangioscopy with direct biopsy for diagnosing challenging biliary lesions.

Endoscopy_UCTN_Code_CCL_1AZ_2AM

## Notes

Endoscopy E-Videos https://eref.thieme.de/e-videos
*E-Videos* is an open access online section of the journal *Endoscopy* , reporting on interesting cases and new techniques in gastroenterological endoscopy. All papers include a high-quality video and are published with a Creative Commons CC-BY license. Endoscopy E-Videos qualify for HINARI discounts and waivers and eligibility is automatically checked during the submission process. We grant 100% waivers to articles whose corresponding authors are based in Group A countries and 50% waivers to those who are based in Group B countries as classified by Research4Life (see: https://www.research4life.org/access/eligibility/ ). This section has its own submission website at https://mc.manuscriptcentral.com/e-videos .

## References

[1] Matsumoto K Kato H Okada H Lymphoid hyperplasia of the bile duct observed on peroral video cholangioscopy Clin Gastroenterol Hepatol 2016 14 e127 e128 10.1016/j.cgh.2016.05.001 27165467

[2] Miyamoto K Matsumoto K Matsubara K lymphoid hyperplasia of the gallbladder extending to the bile duct Intern Med 2023 62 1293 1298 10.2169/internalmedicine.0365-22 36130889 PMC10208782

[3] Muro S Kato H Fushimi H A case of polypoid lesions of the common bile duct observed on peroral video cholangioscopy Dig Liver Dis 2016 48 453 10.1016/j.dld.2015.12.010 26775095

[4] Toru U Mizuno M Ota S Carbon dioxide insufflation is useful for obtaining clear images of the bile duct during peroral cholangioscopy (with video) Gastrointest Endosc 2010 71 1046 1051 20438891 10.1016/j.gie.2010.01.015

